# Assessing the Activity of Individual Group-Housed Broilers Throughout Life Using a Passive Radio Frequency Identification System—A Validation Study

**DOI:** 10.3390/s20133612

**Published:** 2020-06-27

**Authors:** Malou van der Sluis, Yvette de Haas, Britt de Klerk, T. Bas Rodenburg, Esther D. Ellen

**Affiliations:** 1 Animal Breeding and Genomics, Wageningen University & Research, 6700 AH Wageningen, The Netherlands; yvette.dehaas@wur.nl (Y.d.H.); esther.ellen@wur.nl (E.D.E.); 2 Animals in Science and Society, Faculty of Veterinary Medicine, Utrecht University, 3508 TD Utrecht, The Netherlands; t.b.rodenburg@uu.nl; 3 Cobb Europe, 5831 GH Boxmeer, The Netherlands; Britt.deKlerk@cobb-europe.com; 4 Adaptation Physiology Group, Wageningen University & Research, 6700 AH Wageningen, The Netherlands

**Keywords:** tracking, broilers, activity, radio frequency identification, ultra-wideband, video

## Abstract

Individual data are valuable for assessing the health, welfare and performance of broilers. In particular, data on the first few days of life are needed to study the predictive value of traits recorded early in life for later life performance. However, broilers are generally kept in groups, which hampers individual identification and monitoring of animals. Sensor technologies may aid in identifying and monitoring individual animals. In this study, a passive radio frequency identification (RFID) system was implemented to record broiler activity, in combination with traditional video recordings. The two main objectives were (1) to validate the output of the RFID system by comparing it to the recorded locations on video, and (2) to assess whether the number of antennas visited per unit time could serve as a measure of activity, by comparing it to the distance recorded on video and to the distance moved as recorded using a validated ultra-wideband (UWB) tracking system. The locations recorded by the RFID system exactly matched the video in 62.5% of the cases, and in 99.2% of the cases when allowing for a deviation of one antenna grid cell. There were moderately strong Spearman rank correlations between the distance recorded with the RFID system and the distance recorded from video (r_s_ = 0.82) and between UWB and RFID (r_s_ = 0.70) in approximately one-hour recordings, indicating that the RFID system can adequately track relative individual broiler activity, i.e., the activity level of a broiler in comparison to its group members. As the RFID tags are small and lightweight, the RFID system is well suited for monitoring the individual activity of group-housed broilers throughout life.

## 1. Introduction

Knowledge of the relationships between behaviour and health, welfare and performance indicators can be used to assess overall welfare in production animals. Broiler chickens are an example of a livestock species for which insight into these relationships can be valuable. Broilers are, however, often kept in intensive systems, where large numbers of birds (~75,000 per farm in The Netherlands on average; [1]) are housed. This makes it difficult and time consuming to observe the animals and to measure the health, welfare and performance of individual birds. The use of a proxy could potentially help improve the ease with which the health, welfare and performance of the animals can be assessed. A promising trait that could serve as a proxy for multiple indicators of health, welfare and performance is locomotor activity, given the relationship between (changes in) activity and, for example, disease [2], gait problems [3], or body weight [4].

Specifically, information at the individual level is desired to obtain a good view of an individual’s state. Furthermore, when individual data can be collected in a group setting with pen mates present, a more realistic image of an animal’s performance in a production environment can be obtained. In particular, data on individual activity levels of young broilers could be very valuable. For example, it has been established that the activity level of broilers from a fast-growing stock early in life positively correlates with activity later in life [5]. Furthermore, it has been reported that increased activity can result in fewer leg problems [6,7]. Moreover, it has been suggested that increased activity, specifically at an early stage in the growing period, may reduce leg disorders, as leg bone development might be mostly affected in the first days of life (reviewed in [8,9]). Overall, activity levels in the first days of life are potentially very informative for the health, welfare and production of animals later in life and therefore an activity tracking system that allows tracking of individual broilers from the first day of life is desired.

Given that broilers are generally housed in large groups in production systems, identification and activity tracking of individual broilers is a challenge. Sensor technologies, such as computer vision (CV), ultra-wideband (UWB) tracking and passive radio frequency identification (RFID) may offer solutions (see [10] for a review of the applicability of sensor technologies for poultry). In particular, passive RFID seems to have potential for tracking individual broilers from the first day of life, as passive RFID tags do not require batteries and can therefore be small and lightweight [11]. Therefore, in the current study, the suitability of a passive RFID system for tracking individual broiler activity throughout life was investigated.

The implementation of a passive RFID system for tracking individual broiler activity can have added value in comparison to existing tracking systems. For example, several studies on automated activity recording of broilers and its relationship with different health, welfare and performance traits have been performed (e.g., [12]), but many of these studies focused on group-level patterns. For example, Van Hertem et al. (2018) implemented a camera-based automatic animal behaviour monitoring tool (eYeNamic^TM^, Fancom BV, The Netherlands) to study flock activity [3]. They reported a linear trend between flock activity and the average gait score of the flock, where a lower activity level was linked to a worse gait in the flock. Another study, using optical flow patterns, reported, among other things, that a lower average level of flock movement was related to a higher mortality percentage in the flock and that skewness and kurtosis of flock movement were correlated with the percentage of birds in the flock having hock burns [13]. Although such automated flock-level monitoring can be a useful tool for monitoring the overall welfare of a flock, it does not provide information at the individual level and may hereby overlook animals that deviate, which, for example, remain more active than the flock mates and do not show gait problems. Therefore, an automated passive RFID tracking system that provides information on activity levels of individual broilers can have added value. Furthermore, passive RFID has more potential for tracking young broilers in comparison to some other tracking technologies. For example, in our previous study [14], the suitability of an UWB system for tracking individual levels of activity in group-housed broilers was determined. This system was concluded to be suitable for tracking broiler activity, with, among other things, a correlation of 0.71 between video and UWB recorded distances, but it has to be noted that the UWB tags were quite large and heavy, rendering tracking of young broilers, i.e., of less than two weeks old, not feasible [14]. Given the small and lightweight tags of passive RFID systems, passive RFID does have potential for studying birds younger than two weeks old. Passive RFID has already been used for poultry—for example, to study range use, nest box use and feeding behaviour (e.g., [15,16,17]; reviewed in [10]). Passive RFID has also been used to study general locomotor activity [18]. In a study by Kjaer (2017), RFID antennas were placed inside the central area of the pen of the birds in order to record the locomotor activity of laying hens [18]. These antennas covered an area equal to 33% of the total pen area. Possibly, a more detailed image of activity can be obtained using more antennas, covering a larger percentage of the pen.

In this study, the suitability of a passive RFID system, covering the full pen, for tracking individual broiler activity throughout life was investigated. The two main objectives of this study were (1) to validate the RFID recorded locations by comparing these to the locations recorded on video, and (2) to assess whether the moving distances calculated from the RFID data, using the recorded antenna positions over time and the distances between antennas, could serve as a measure of activity. To this end, the RFID recorded distances were compared to the total distance moved as recorded on video and using a validated UWB tracking system.

## 2. Materials and Methods

### 2.1. Ethical Statement

Data were collected under control of Cobb Europe. Cobb Europe complies with the Dutch legislation on animal welfare. This study is considered not to be an animal experiment under the Law on Animal Experiments, as confirmed by the local Animal Welfare Body (20th of June, 2018, Lelystad, The Netherlands).

### 2.2. Location and Subjects

This study was performed on a broiler farm in The Netherlands. In total, 40 male broiler chickens from two genetic crosses were used. The birds were fitted with RFID tags on the day of hatching and four birds, two from each cross, were marked visually using marker spray in four different colours. Near-continuous RFID recordings were made from day of hatching to 34 days old. At 15 days old, birds were also fitted with an UWB tag. Due to limitations in the number of tags, a total of 34 out of 40 birds were fitted with an UWB tag.

### 2.3. Housing

The broilers were housed in a rectangular pen, with a size of approximately 1.8 by 2.6 m and an area of approximately 4.7 m^2^. In this pen, feed and water were provided ad libitum. Wood shavings were provided as bedding. The birds were kept under a commercial lighting and temperature schedule and were vaccinated according to common practice [19].

### 2.4. Radio Frequency Identification (RFID) System

**Figure 1:**
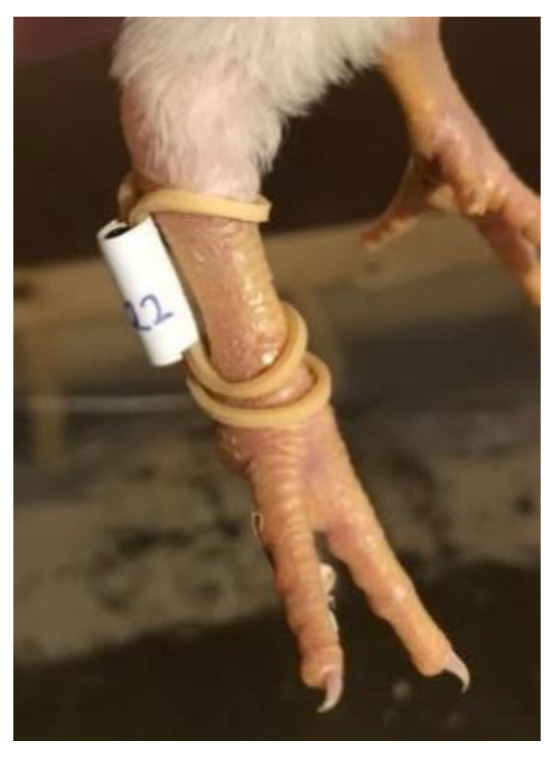
Radio frequency identification (RFID) tag fitted to a broiler’s leg using a rubber band (shown in the larger size) and tape.

To track individual broilers and monitor their activity throughout life, a passive RFID system from Dorset Identification (Dorset Identification B.V., Aalten, The Netherlands) was used. All 40 broilers were fitted with a high frequency (HF) tag operating at 13.56 MHz. These tags had a size of approximately 15 × 3.7 mm and a weight of less than one gram. The tags were fitted to the legs of the birds using rubber bands and tape (Figure 1). The rubber bands were changed to a larger size once during this study in order to fit the birds’ legs, and checked every couple of days.

**Figure 2:**
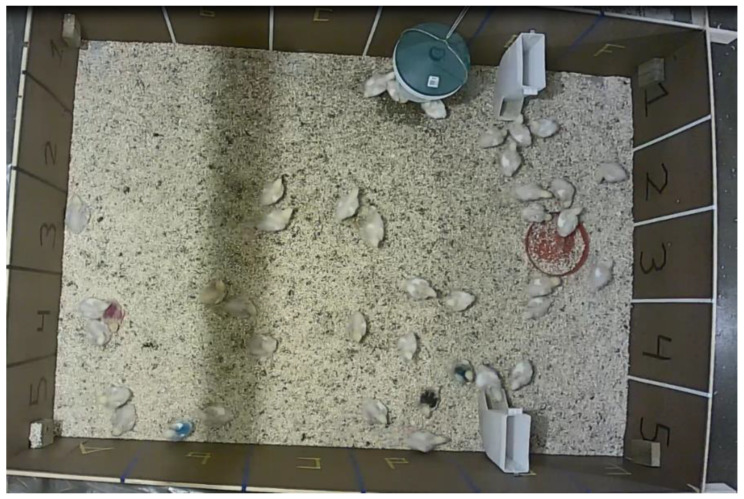
Top view of the tracking pen during this study, with bedding on the false floor underneath which the RFID antennas are situated. Lines on the pen walls indicate the position of the antenna grid.

The pen in which the broilers were housed was fitted with 30 HF antennas of 32 by 41 cm, in a grid covering the full pen (Figure 2). The antennas were located on the underside of a false floor in order to protect them from water, moisture and dirt. The antennas were connected to one of two readers (HF RFID reader DSLR1000, freaquent froschelectronics GmbH, Graz, Austria) that read the tags at the antennas and sent the information to the custom-made RFID software from the same company. The tags could generally be read in the 32 by 41 cm rectangle that each antenna covered and up to a height of approximately ten centimetres. A log file was stored which included the time of registration, the unique hexadecimal ID code of the tag and the location, i.e., antenna, at which the tag was registered. Antenna 7 of the RFID system (see Section 2.6.1. RFID Distance Calculations) was not recording birds due to a technical issue. The recording frequency used here was generally one registration per second for each antenna. In the instances in which antenna switches occurred within the same second, two different registrations could be observed. RFID recordings were made continuously for 24 h per day, with exception of the periods in which the leg bands were being checked or the birds were being weighed. Weighing was done four times during the trial and took approximately two to four hours. This included checks of the leg bands, and UWB tags when applicable. Additional leg band and UWB tag checks were performed three times during the growing period and took up to two hours each time. Two broilers died during this study and for two broilers there were too much missing RFID data (<75% of data available) due to loss of their leg tags during this study. The final sample size was 36 broilers—of which, 33 had an UWB tag. The weights of the broilers over time are shown in Table 1.

### 2.5. Study A: RFID Location Validation

**Figure 3:**
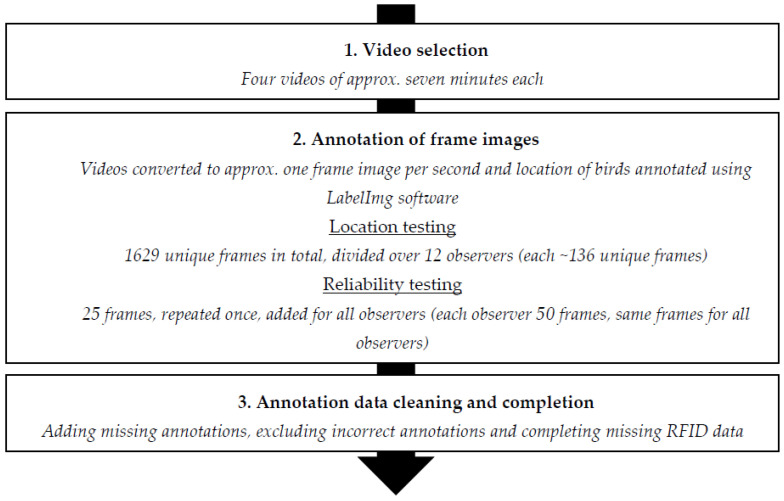
Graphical overview of the implemented approach for the RFID location validation study.

To study whether the RFID system correctly registered the location of individual birds over time, the RFID log file was compared to video recordings. The implemented approach is visualised in Figure 3.

#### 2.5.1. Video Selection

Top-view video recordings were made for approximately two hours per day, using a Zavio B6210 2MP video camera (Zavio Inc., Hsinchu City, Taiwan) with a frame rate of 25 frames per second. From these recordings, four videos with a duration of approximately 7 min each were selected at random: one from 0 to 6 days old, one from 7 to 12 days old, one from 17 to 26 days old and one from 28 to 34 days old. These periods were selected because of the differences in activity, body size and absence/presence of UWB tags between these periods, and excluded days closely after fitting the UWB tags or when not all data were available. The details of the four videos are shown in Table 2. Before placing bedding and birds in the pen, an image showing the RFID grid was taken. This image was used as a reference for the location of the birds in order to score coordinates and associated areas of the different antennas.

#### 2.5.2. Annotation of Frame Images

The randomly selected video recordings were converted to frame images, with a rate of approximately one frame per second, using VLC media player (VideoLAN, Paris, France). In these frame images, four colour-marked birds were annotated using LabelImg software [20]. The frames were annotated by in total twelve different observers, all scoring a unique set of one-twelfth of the total number of frames (i.e., ~136 out of 1629 frames), as well as an overlapping set of frames. This overlapping set of frames consisted of 25 frames, randomly taken from the overall 1629 frames available, that were repeated once, resulting in 50 frames that were the same for every observer. This overlapping data set allowed for calculation of inter- and intra-observer reliability, in which 100 annotations, i.e., four animals from 25 frames, could be compared and missing annotations were deleted row-wise. For the inter-observer reliability, 85 annotations were left for comparison between all observers after removal of missing annotations. The number of annotations available for intra-observer calculations after removal of missing annotations differed per scorer and ranged between 83 and 98.

#### 2.5.3. Annotation Data Cleaning and Completion

The annotations of the unique sets of frames from all observers provided coordinates of the location of the birds in the image. These coordinates were compared to the location of the different antennas from the reference image in order to assign an antenna number to the location of the birds. Sometimes, a scorer missed a bird—for example, because it was hidden or simply overseen, —or labelled two different birds with the same ID. In these instances, the correct annotation was added later on, by a single scorer who was already included in the group of annotators. It is important to note that this step was only performed for the frames included in the location validation set and not for the frames used for determination of inter- and intra-observer reliability, where only raw data were used. When a bird was positioned at the side of the pen, leaning against the pen wall, it could happen that the bird was annotated outside the antenna range. In these instances, a missing value was given and this observation was excluded from the calculations. Upon visual inspection of the coordinate data, some incorrect annotations were observed, where birds were located for only a single frame at a non-adjacent antenna. These incorrect annotations were removed, resulting in a missing data point. Furthermore, if location 7, where the antenna was not functioning, was scored on video, this value was replaced with a missing value and not included in the analysis. The final output data from the videos that were used here included a time stamp, the animal ID and the antenna at which the animal was located. This output was then compared to the output of the RFID system, which included the same variables. For this location validation, the output of the RFID system was manually completed after recording, as the RFID system sometimes did not register birds when they were lying down, resulting in missing recordings. The RFID system functions best when the tags are in a vertical position, i.e., at a 90 degree angle in comparison to the antenna floor, which is the case when birds stand. When birds are lying down, the tags are in a position parallel to the antenna floor and are often not registered. It was assumed that as long as a bird was not registered elsewhere, the bird was still located at the antenna position where the bird was last registered. This assumption was used to fill in the missing rows in the RFID data for the location comparison. In later analyses of distances moved, and future implementations, this is not necessary, as the main factor of interest in those cases are likely the antenna switches, and when studying these the last recorded position can be used, regardless of the time between the last recorded position and the next recorded position. However, for the location validation this would result in missing data, and therefore missing rows were filled in based on this assumption. Both the overall registrations and the trajectory walked by the birds were compared between the RFID system and video recordings. For the overall registrations, the percentage of (dis)agreement was calculated, while for the trajectory analysis the path of the animals was visually compared.

### 2.6. Study B: Moving Distance Validation

**Figure 4:**
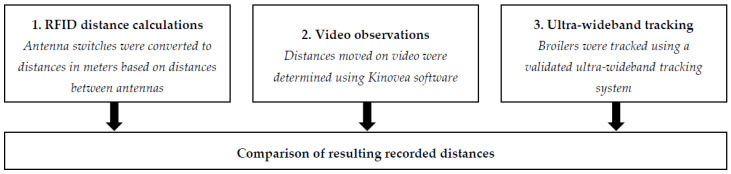
Graphical overview of the implemented approach for the RFID distance validation study.

To study whether the distance moved as recorded with the RFID system was a good indicator of activity, the RFID output, in terms of antenna switches per unit of time, was compared to the distance moved that was recorded (1) from video and (2) using an UWB system. The implemented approach is visualised in Figure 4.

#### 2.6.1. RFID Distance Calculations

**Figure 5:**
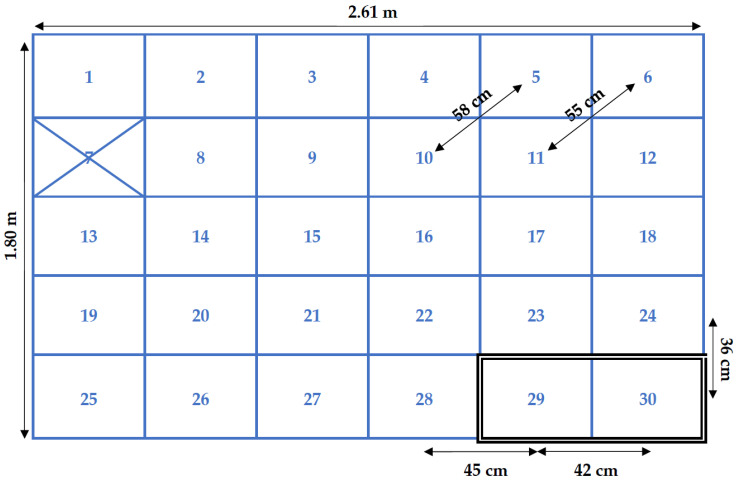
Schematic top view of the RFID grid. One of the fifteen PVC antenna panels that includes two RFID antennas is outlined as an example (antenna 29 and 30). Distances between centre points of antennas are indicated, as well as the total size of the grid. The non-functioning antenna is marked with an X.

The RFID recordings required conversion to metrical distances. The number of switches between antennas was used as an initial measure of activity for the RFID system. The RFID antennas were all the same size (32 × 41 cm), but were not exact squares, so a correction was made in the RFID output to account for the difference in distance between centre points of antennas in the x and y direction. Furthermore, antennas were mounted on a total of fifteen PVC panels, in sets of two with identical configurations and the short sides of the antennas adjacent to each other, resulting in a difference in distance between two antennas on the same panel and between adjacent antennas on different panels. This was also accounted for in the RFID distance calculations. Figure 5 shows a schematic top view of the antenna grid. The shortest distance between two centre points was 36 cm, and this distance was set as ‘one switch’. All other switches were calculated in relation to this distance, e.g., a distance of 45 cm was recorded as 1.25 switch. For each antenna switch, the distance between the last recorded and next recorded antenna was calculated using the shortest possible route to this antenna, i.e., a straight line. False switches could occur when an animal was on the boundary between two antennas, which could result in alternating registrations at the two adjacent antennas. Therefore, if an animal was registered at a new antenna and this same new antenna was also registered less than five seconds before, the switch in between was not included in the calculation of the total number of antenna switches. Per tracking period, the antenna switches were added up as an indicator of the activity level of that animal in that period and could be multiplied by 0.36 to convert this to a distance in meters.

#### 2.6.2. Video Observations

For the video comparison, Kinovea software (version 0.8.15; [21]) was used to calculate the distances moved by four colour-marked birds. The length of one side of the tracking pen was used for calibrating the distances. The positions of the birds were marked in the video by drawing a circle around each bird, closely matching the outline of the lateral sides of the bird. This was done to make it easier to see when a bird changed position and to set a threshold for movement. In this study, movement was only included if the bird shifted at least half a body width, i.e., the centre of the bird moved outside the circle. When this criterion was met, the bird was marked with a tracker in the video and the movement of the broiler was tracked automatically. Manual corrections were applied when the tracking algorithm did not recognise the movement of the bird or tracked the path incorrectly. When a bird stopped moving, the recording was stopped and the distance covered was noted, before placing the circle around the bird again and resuming the video analysis. After completion of the video analyses, the distances per animal and per time period were summed in order to provide the full distance moved by each animal in the desired timeframe. Of the two-hour video recordings each day, approximately one hour of video was analysed per day for a total of eight days, ranging from 25 to 33 days old. Day 27 was excluded as no RFID data were available due to a technical error. In these videos, the distances moved of the four colour-marked birds were analysed, resulting in four data points per one-hour video. Due to one colour marking fading away, three one-hour videos were analysed for three instead of four animals. A total of 29 one-hour records was available for analysis. The distances recorded in these videos were compared to the distances recorded with the RFID system during the same periods.

#### 2.6.3. Ultra-Wideband Tracking

For the comparison with UWB tracking, a Ubisense UWB system with Series 7000 sensors and compact tags (Ubisense Limited, Cambridge, UK) was used, in combination with TrackLab software version 1.4 (Noldus Information Technology, Wageningen, The Netherlands). In total, 34 out of 40 birds were fitted with an UWB tag, due to a limitation in the number of tags available during this study. These tags had a size of 3.8 by 3.9 cm and a weight of approximately 25 g, and were fitted to the birds using elastic bands around their wing base. This UWB system, the setup and validation are described in more detail in [14]. A sampling rate of one sample approximately every 6.91 s was implemented. The UWB system recorded the activity of the birds near continuously from 15 days old, with exception of the periods in which the leg bands were being checked or the birds were being weighed. The UWB output that was used here was the total distance moved in meters per individual per tracking period, where the tracking periods were selected to match the duration of the analysed videos and corresponding RFID recordings, with a possible shift of several seconds due to the sampling rate of the UWB system. Furthermore, to study whether longer RFID and UWB recordings would average out any shorter-term differences, the average hourly activity levels from the RFID and UWB systems were calculated, per day and for each animal, and were corrected for the recording duration. Because we ran out of batteries during the experiment and because of too much missing UWB data (<75% of data available), near-complete UWB data was available for 25 animals. In total, approximately 18 days with UWB and RFID data were included in the comparison between UWB and RFID recorded distances.

### 2.7. Statistical Analyses

All statistics were performed using R version 3.5.0 [22]. To validate the location output of the RFID system, a comparison with the locations recorded on video was made. To study the inter-observer reliability of the annotated video recordings, a Fleiss’ Kappa calculation was performed on one-half of the data (i.e., no repetitions of the same frames within an observer), using the irr package [23]. To determine the intra-observer reliability of the annotation of the video recordings, Cohen’s Kappa tests from the irr package were performed. Descriptive statistics were used to study the number of matches and mismatches between video and RFID in terms of location where the birds were observed. Visual comparisons were made of the trajectories walked by the birds. To assess whether the antenna switches recorded per unit time could serve as a measure of activity, Spearman rank correlations were determined between the distances recorded by the RFID system, on video and as recorded with the UWB system, as the data were not normally distributed. The 95% confidence intervals for the Spearman rank correlations were calculated by bootstrapping using the RVAideMemoire package [24]. Strictly speaking, the data points in the correlations are non-independent, as the same animals have been repeatedly measured across days. To determine the common within-day or within-individual correlation, repeated-measures correlations were implemented [25] using the rmcorr package [26]. This analysis assumes, among other things, linearity of the relationship between the two variables and normal distribution of the errors, and the assumptions were not fully met here. Therefore, interpretation of these results requires some caution. However, the resulting correlational values fell within the 95% confidence intervals of the overall correlations and are therefore not reported in the results. A Spearman rank correlation was also used to determine the correlation between the hourly average activity as recorded by the RFID and UWB system. Furthermore, for the analysis of the hourly average activity as recorded by the RFID and UWB system, repeated-measures correlations were again implemented to determine the common within-day or within-individual correlation, and are reported in the results. Again, the assumptions were not fully met and the interpretation of these results requires some caution. Visualisations were made using the trajr [27], ggplot2 [28], ggpubr [29] and rmcorr [26] packages. The level of statistical significance was set at 0.05. In the text, reported results are rounded to two decimals.

## 3. Results

### 3.1. Study A: RFID Location Validation

The overall inter-observer reliability for the annotated antennas was 0.90 (Fleiss’ Kappa, subjects = 85, raters = 12, Z = 209, *p* < 0.001). The intra-observer reliability for the annotated antennas was on average 0.92, ranging from a minimum agreement of 0.75 to a maximum agreement of 0.99 (see Table S1 in the supplementary data). Comparison of the locations on video and from the RFID system showed that video 2 had the highest exact agreement with RFID (80.9% matches), while video 3 had the lowest exact agreement (47.4% matches; Table 3). Overall, 62.5% of the registrations exactly matched between RFID and video. This percentage increased to 99.2% when allowing for a deviation of one antenna, i.e., a registration at one of the maximum eight neighbouring antennas.

**Figure 6:**
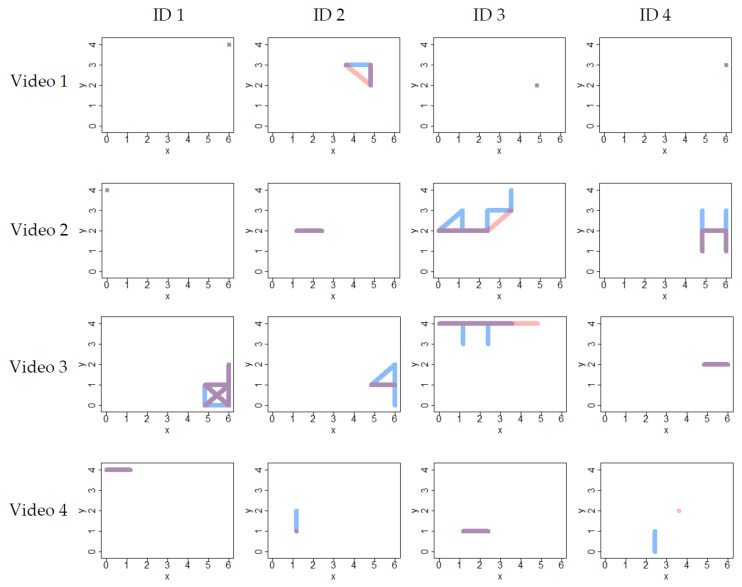
Trajectories walked by individual birds in the four videos. Each square represents the pen from above. The four videos (see Table 2) are on separate rows, while different columns represent different individuals in these videos. The blue line represents the video track, while the pink line represents the RFID track. Overlapping parts of the track are depicted in purple.

**Figure 7:**
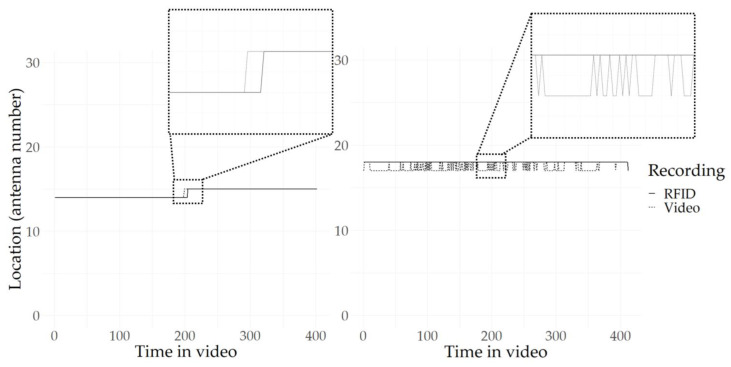
Locations over time for two individuals in two videos. The solid black line represents the RFID location, while the dashed black line represents the video location. In the boxes, zoomed-in parts of the graph are shown. (**a**) Almost fully matching track from animal ID 2 from video 2; (**b**) track with many mismatches occurring from animal ID 4 from video 3.

Although there appeared to be more detail, or positional changes, in the video data compared to the RFID data, the trajectories walked by the birds according to the RFID system and as observed on video showed good agreement (Figure 6). Moreover, the RFID location of the individuals did not deviate strongly from the location on video. In other words, no RFID registrations were observed in regions where the animals were not near. However, although the trajectories from video and RFID matched well, the timing of the recorded location switches sometimes differed between video and RFID. To illustrate this, Figure 7 shows the video and RFID locations over time of two example tracks, one from video 2 (high percentage of matches; animal ID 2) and one from video 3 (low percentage of matches; animal ID 4). In both these examples, the animals were not very active and the RFID and video trajectories matched completely (Figure 6). However, Figure 7a shows that the video and RFID matched almost completely for the first of the two tracks, while Figure 7b shows that the video registered many switches between the two recorded antennas for the second track, causing mismatches between RFID and video in terms of location for part of the recording.

### 3.2. Study B: Moving Distance Validation

**Figure 8:**
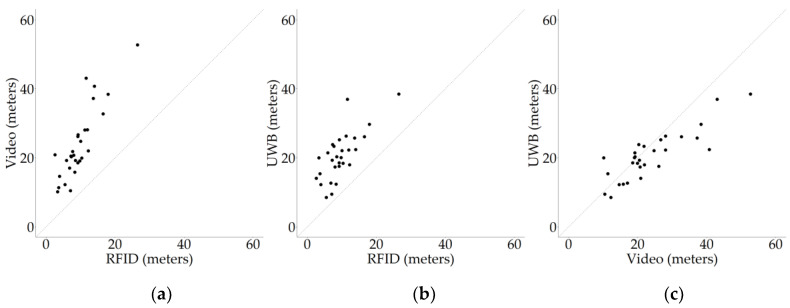
Scatter plots of the relationships between two measuring techniques. Distances are depicted in meters on both axes. The dashed line indicates the diagonal. Dots represent individual data points. (**a**) RFID versus video; (**b**) RFID versus UWB; (**c**) video versus UWB.

There were moderately strong Spearman rank correlations in terms of distances moved between RFID and video (r_s_ = 0.82 (95% CI 0.61–0.92), S = 730, *p* < 0.001; Figure 8a), between RFID and UWB (r_s_ = 0.70 (95% CI 0.41–0.86), S = 1224, *p* < 0.001, Figure 8b) and between video and UWB (r_s_ = 0.79 (95% CI 0.53–0.92), S = 854, *p* < 0.001; Figure 8c).

**Figure 9:**
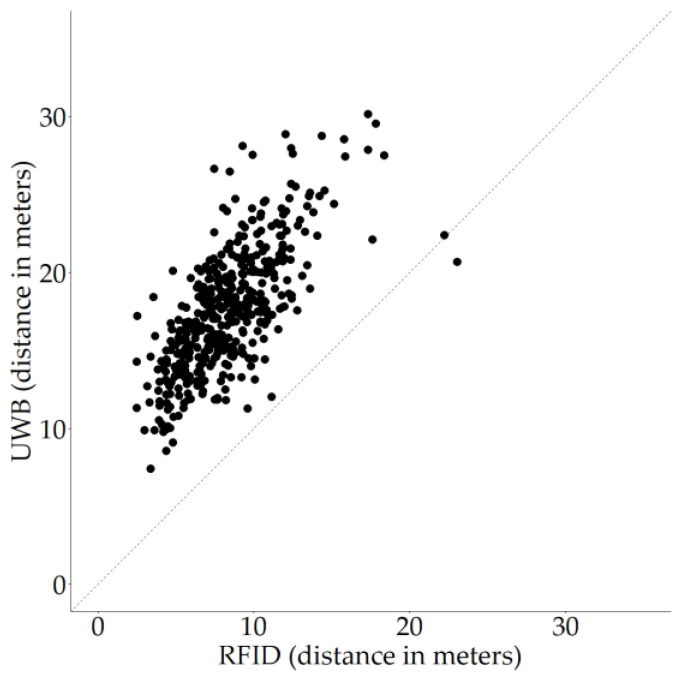
Scatter plot of the relationship between RFID and UWB over a longer period of time, as average distances moved per hour for each day and animal. Distances are depicted in meters. The dashed line indicates the diagonal. Dots represent individual data points.

**Figure 10:**
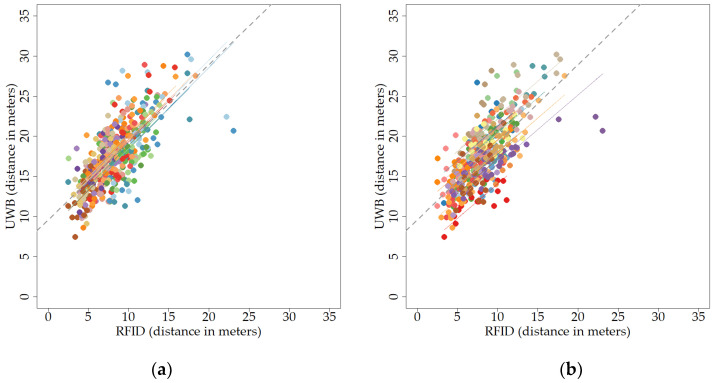
Scatter plot of the relationship between RFID and UWB over a longer period of time, as average distances moved per hour for each day and animal, looking at the correlation within days (**a**) or individuals (**b**). Different colours represent different days (**a**) or individuals (**b**). Distances are depicted in meters. The dashed line indicates the regression when ignoring the repeated-measures variable, i.e., indicating the overall correlation. Dots represent individual data points. (**a**) Scatter plot of the relationship between RFID and UWB for different days; (**b**) scatter plot of the relationship between RFID and UWB for different individuals.

There was a moderately strong Spearman rank correlation between RFID and UWB when using the average distance moved per hour, calculated per day and animal (r_s_ = 0.71 (95% CI 0.65–0.75), S = 4041800, *p* < 0.001; Figure 9). However, the UWB system nearly always recorded higher distances than the RFID system (Figure 9). When looking within days, a correlation of 0.65 was observed (repeated-measures correlation, r = 0.65 (95% CI 0.59–0.70), df = 417, *p* < 0.001; Figure 10a). When looking within individuals, or tags, a correlation of 0.73 was observed (repeated-measures correlation, r = 0.73 (95% CI 0.69–0.78), df = 410, *p* < 0.001; Figure 10b). The repeated-measures correlations strongly resembled the overall correlation without taking repeated-measures into account (Figure 10).

## 4. Discussion

In this research, it was studied whether a passive high frequency RFID tracking system was suitable for monitoring the individual levels of activity of broilers throughout life. On average, 62.5% of the RFID recordings matched exactly with video in terms of the location of the animals and 99.2% of the registrations matched when allowing for a deviation of one antenna, indicating that the RFID system can give a good approximation of the location of individual birds. Furthermore, distances recorded with the RFID system correlated well with both video and UWB measurements. These correlations indicate that the RFID system can provide good estimations of the activity levels of individual broilers in relation to their pen mates.

### 4.1. Study A: RFID Location Validation

Overall, almost two-thirds of the location registrations exactly matched between RFID and video. Furthermore, the difference in position detected with the RFID system and on video was rarely larger than one antenna, i.e., birds were rarely registered further away than an adjacent antenna compared to the position on video, as indicated by the 99.2% near matches. This indicates that the RFID system can give a good approximation of the location of individual birds and has potential to be implemented to, for example, study locational patterns of individual birds over time. However, there was some disagreement between video and RFID in terms of exact registered locations, which has several possible explanations that are related to how RFID and video differ in the way they can be used to detect the birds. These include the reference point used for location determination, the level of detail of the recordings, the effect of intermittent tag registrations, and the tag orientation, and are outlined below.

#### 4.1.1. Reference Point for Location Determination

On video the centre point of the birds is used to determine the location of the bird, while the RFID tag is attached to a single leg. This could result in a bird being registered at a different antenna on video than with the RFID system, as the centre point of a bird might be situated at a different antenna than the leg of the bird. It has been reported that broilers generally place their feet in a lateral position relative to the hip [30]. In particular, when birds are larger, this may affect the results of the position recordings on video and using RFID. A positive relationship between body weight and pelvis width, measured across the back of a standing chicken as the distance between the exterior of the thighs, has been observed in chickens [31]. Therefore, the absolute distance between the centre point of the birds and their legs may increase further as the birds grow, which could explain why more mismatches were seen in video 3 and 4, in which the birds were larger. However, it must also be noted that video 3 and 4 included birds with UWB tags, while video 1 and 2 did not. With the current data, it cannot be completely ruled out that the UWB tags affected the RFID recordings, but given that the two systems communicate on different radio frequencies, this is not expected. It is therefore hypothesised that the difference in size of the birds was the main cause of the higher number of mismatches in video 3 and 4 and likely, if on video the leg of the bird that was fitted with an RFID tag was annotated, fewer mismatches between RFID and video would be observed. However, with the current setup using top-view video recordings, it was not feasible to annotate single legs of birds as these were not always visible. Therefore, the centre point of the birds was used as an approximation, likely resulting in an increase in mismatches between RFID and video.

#### 4.1.2. Level of Detail of the Recordings

As was shown in Figure 7b, there were more location switches registered on video, compared to with the RFID tracking. One possible explanation for this higher number of switches on video is that the video recorded more detailed changes than the RFID system. For example, when a bird was situated on the edge between two antennas, even a slight shift of a few pixels could result in the bird being registered at a different antenna than before. This shift might be registered on video due to a slight movement of the birds, but can also be caused by an inaccuracy in the annotations of the birds. Moreover, at the very edges of the antenna panels, there was not always full antenna coverage, as there was some uncovered space between the individual antennas. Therefore, when the video registered a bird at the edge of a new antenna, the RFID system was not always able to ‘see’ this bird and did not register a new location for this bird.

#### 4.1.3. Intermittent Tag Registrations

In some cases, the RFID system did see the tag when it was situated between two antennas. This could result in the bird being registered in two positions intermittently, while the bird was not moving on video, resulting in approximately half of the registrations being a mismatch. In the distance validation, this was filtered out in order to exclude false positives for locomotor movement between antennas, but in the location analysis these switches were kept to allow a full second-by-second comparison of the positions of the animals using the raw RFID data as a basis. Such intermittent registrations at two antennas were observed in the current study and have also been reported in other studies using RFID technology to track positions of animals (e.g., low frequency RFID in rats [32]; ultra-high frequency RFID in mice [33]).

#### 4.1.4. Tag Orientation

The RFID system sometimes could not register birds when they were lying down. The RFID system functions best when the tags are in a vertical position, i.e., at a 90 degree angle in relation to the antenna floor. This is often the case when birds stand upright. When birds are lying down, the tags are in a position parallel to the antenna floor. Therefore, when a bird shifts position without fully rising to stand, the RFID system may not register this movement. In the analysis performed here, missing RFID registrations were completed using the last recorded position of the animal, because no ‘new’ information was available from the RFID system and therefore the last registered antenna was the most likely current position of the animal. However, this position may potentially no longer have been the correct one, for the reason mentioned above. For practice, the notion that birds are often not registered when lying down is not a cause for concern. On the contrary, when one is interested in recording locomotor activity, it might even be preferable to not register movement of birds that are not standing up, as this may not be considered as true locomotor movement of the bird. By not registering birds while they are lying down, likely no relevant information is lost and it might reduce noise in the data that could be caused by birds slightly shifting position, for example, because they are preening, while lying down.

Altogether, the differences in the way RFID and video register the birds may underlie the mismatches that are observed. However, the trajectory plots (Figure 6) show that when RFID and video do not agree on the trajectory, the deviation is generally not larger than one antenna. In other words, birds were, at least in these videos, never registered in locations where they really could not be located near, i.e., at far-away antennas in relation to the position on video. It appears that the RFID system might not always register all changes that are happening on video, because these changes might be too subtle for the RFID system to detect or are not actually caused by birds walking. However, the RFID system also does not register birds in locations where they are not located near based on the video observations. Overall, it appears that the RFID system is not very sensitive to small changes, but does provide a good approximation of the location of birds over time.

### 4.2. Study B: Moving Distance Validation

#### 4.2.1. RFID Versus Video

In this study, it was assumed that the distance moved as recorded from video is the true distance moved by the animals. There was a moderately strong rank correlation (0.82) between video and RFID in terms of distance moved. This indicates that the RFID system is suitable for comparison of activity levels between animals, and can be implemented to study individual differences in activity levels in a more time-efficient and objective manner compared to visual observations from video recordings. In terms of absolute distances moved, the distance as recorded from video was generally somewhat higher than the distance registered by the RFID system. There are several explanations for this finding.

First, the RFID and video recordings could differ in the exact starting point of the distance recording. In theory, the RFID and video recordings spanned the same time period, but, as explained earlier, the RFID system did not always register birds, especially when they were lying down. Consequently, when a recording started, the RFID system might only have registered a bird when something in the position of the bird changed—for example, when it moved to a new antenna, as it was possibly no longer registering the previous position of the bird. This could result in the first ‘switch’ between antennas being missed, as only the second antenna might be registered, while on video, all movements from the start of the recording can be observed. Consequently, the RFID system might show a slightly lower recorded distance compared to video.

Second, as discussed earlier, the video can capture much smaller changes than the RFID system. On video, all changes can be observed, while the RFID system only registers movement between antennas, and not within antennas, which can cause the distance recorded with the RFID system to be lower. The absolute difference between the recorded distances on video and using RFID appeared larger when birds moved longer distances (Figure 8a). Redfern et al. (2017) used a low frequency RFID tracking system in rats, with a setup similar to the setup in the current study, and compared RFID and manual distance tracking [32]. They found a good correlation between the two (ICC = 0.83), although it depended on the position of their implanted RFID tag, but noted that the RFID distance was lower than the manual tracking for higher activity levels. One of their explanations for this discrepancy was that faster locomotion could have impacted the tracking accuracy [32]. Indeed, other studies have reported that when tags move faster, their probability of registration can decline [34]. In the current study, this may have impacted the recorded distances in two ways. First, some movement to and from antennas might be missed due to unrecorded antenna visits, resulting in a lower recorded RFID movement, and second, via the way the RFID distances were calculated. The RFID moving distances were based on the shortest possible straight line from one antenna to the other (see Figure 5). It is, however, unlikely that animals always walk in a straight line from one antenna to another. Therefore, a part of the true distances moved may be missed by using the shortest possible route in the calculations. This influences the recorded distances between antennas that are not adjacent. Specifically, when an animal is registered at two subsequent antennas that are far apart, the absolute distance underestimation can become large, as the absolute difference between the calculated shortest route and possible true routes is especially large here. However, although in the current study the absolute difference between RFID and video was observed to be larger at higher distances, the relative difference appeared to remain relatively constant. Furthermore, the earlier-mentioned studies reporting an effect of movement speed of the tag on recording reliability implemented low frequency RFID, while in the current study, high frequency RFID was implemented, which has a faster read rate [35]. Therefore, it is less likely that fast movements may have been missed in a similar way, and it appears more likely that the distances moved within the antennas are the main cause of the difference between RFID and video, as opposed to the moving speed of the birds.

Third, during this study, antenna 7 of the RFID system was not functioning and therefore not recording birds. Moreover, after this study, it was discovered that the antennas close to the drinker were no longer recording birds near the end of the tracking period, likely due to the moisture in the bedding underneath the drinker blocking the RFID signal. This could have resulted in some missed registrations, and hereby could have lowered the recorded RFID distance in comparison to video.

Overall, the distance moved calculated from the RFID data may not exactly represent true distances moved. However, the moderately strong correlation between video and RFID does allow comparison of activity levels between animals tracked with the same RFID setup.

#### 4.2.2. RFID Versus UWB

There were moderately strong rank correlations between UWB and RFID in the one-hour sessions (0.70) and in the longer recordings (0.71). This indicates that the RFID system and the UWB system generally agree on the relative ranking of animals in terms of distances moved. Whether animals are tracked using the RFID system or the UWB system, the same animals will likely be identified as relatively active or inactive by both systems. In terms of absolute distances, the distance recorded with the UWB system was higher than the distance recorded with the RFID system for nearly all data points. This could again be explained by the RFID system not registering movement within antennas, while the UWB system can theoretically pick up on all movements. However, the difference between RFID and UWB may be enlarged by the localisation error or noise that may occur with the UWB system. There may be some noise in the localisation of the UWB system, which could result in slight deviations from the actual position of the bird over time, hereby increasing the recorded distance moved [14]. The repeated-measures correlations on the longer-period UWB and RFID data showed that the distribution of data points differed somewhat between days and individuals. The differences in distribution of data points between individuals can be explained by differences in activity between birds, i.e., it can be expected that some birds are consistently more active than others or move faster, or in a different way, compared to others, which may affect the RFID and UWB recordings to some extent. The difference in distribution of data points between days is less intuitive. Although differences in overall activity levels are expected across days, theoretically the UWB and RFID systems function in a consistent manner and should therefore not show differences in the relationship between them. However, the systems can be affected by the surroundings. For example, metals or water in the environment may affect RFID systems [35], while UWB systems may miss registrations when tags are in a (partially) covered position (e.g., [36]; also for a discussion on how to deal with missing UWB registrations). In this study the surroundings were kept the same as much as possible, so it would be expected that the influence of water, metals and coverage of tags was the same across the entire trial and therefore the differences between days may simply reflect random noise. However, as mentioned earlier, the antennas close to the drinker were no longer recording birds near the end of the tracking period, likely due to the moisture in the bedding underneath the drinker blocking the RFID signal. This could have reduced the RFID recorded distances over time, and hereby the correlation between UWB and RFID. Regardless, as is shown in Figure 10, the observed correlations did not differ much between the repeated-measures and assumed-independent data and the direction of the relationship between RFID and UWB appeared to be the same. In practice, the overall correlation across days and individuals seems the most relevant, as this is the correlation that is representative of comparing animals that are tracked at different timepoints, regardless of whether these same animals have been tracked before and on what specific day. It is important to note, however, that an approach like this does not take changes in activity over time within animals into account, so it would be advisable to only compare the activity of animals that are approximately the same age at the time of measurement. Overall, although there are differences between the two tracking systems in observed absolute distance moved, it appears that the RFID and UWB systems show good agreement on which animals are relatively active or inactive.

#### 4.2.3. Video Versus UWB

There was also a moderately strong correlation between video and UWB, with r_s_ = 0.79. This indicates that the UWB recorded activity reflected the ranking of the animals in terms of distances moved well. In our previous study [14], a similar approach was used and a correlation between video and UWB of 0.71 was reported. The correlation in the current study was somewhat higher, but it must be noted that the confidence interval for the correlation of 0.79 ranges from 0.53 to 0.92 and thus includes the earlier reported correlational value. Therefore, no strong conclusions can be drawn from this difference between studies. It is noteworthy, however, that in the current study the observations were made later in the life of the birds. In our earlier study [14], the observations were made from 15 to 33 days old, while in the current study the observations were made from 25 to 33 days old. Given the decreasing trend in activity that is often reported as broilers age (e.g., [14,37,38]), it was likely that in the current study somewhat lower average activity levels were included in the comparison between video and UWB, compared to the earlier study. However, the median distance moved on video, assumed to be the true distance moved, observed in the earlier study was approximately 20 m (ranging from approximately 2 to 57 m in this study), while in the current study this was approximately 21 m (ranging from approximately 10 to 53 m). The setup of the current study did, however, differ in comparison to our earlier study in terms of, among other things, the placement of water and food, the density of the birds and the human scorer of the videos. Possibly, this may have influenced the observed correlation, but this could not be confirmed with the data from the current study. Generally, however, it is important to note that the distance comparisons in this study, with exception of the RFID-UWB comparison over longer periods of time, were made using data from 25 to 33 days old, and therefore the observed correlations are representative only of the later part of broiler production. Although it is not expected that the relationships between systems are very different at different timepoints, additional work involving RFID early in life is needed to confirm equal performance of the RFID system at younger ages.

Overall, the moderately strong correlations between the different observation methods suggest that the RFID system and the UWB system are both suitable for estimating differences in activity levels between individual birds. However, as the RFID system allows for monitoring activity from hatching onward, due to its small and lightweight tags, it is here deemed to be the preferable method for tracking individual activity in group-housed broilers throughout life.

### 4.3. Future Prospects for Implementation of the RFID System

The RFID system that was implemented here shows great potential for recording individual activity levels of broilers throughout life, on multiple animals simultaneously, in a time-efficient and objective manner. The current setup provided a high level of detail, using antennas with a size of 32 by 41 cm, and can be of great value in research into behaviour of group-housed birds. However, as mentioned earlier, some difficulties were experienced with the RFID system. One antenna was not recording animals due to a technical error that was related to a faulty contact in the antenna. Further, several antennas were not recording birds at the end of the trial due to moisture in the bedding underneath the drinker. In general, the RFID system can be sensitive to the environment, which is important to take into account when implementing the system in practice. Furthermore, the RFID system used here is relatively easy to dismantle and move to a different location, but it would be advisable to setup the system in a location where it can remain for longer periods of time, as the transport and change in surroundings can result in recalibration of antennas being required for the system to work as desired and cleaning of the antennas is difficult as no water can be used.

The intended purpose of implementing an RFID system is important to consider when selecting a system, as this may affect the requirements for the RFID system. For example, the current setup implemented high frequency RFID, among other things, to allow registrations of multiple animals at an antenna and avoid data collision. However, the radio frequency also has consequences for how much the system is affected by water and metal in the environment and what communication ranges can be achieved [35], and it is therefore important to weigh up the benefits and downsides when selecting a system. The level of spatial resolution can also vary, depending on the antenna size and configuration. For more detailed position recording, more and smaller antennas may be desired. However, for larger scale implementations in practice, a high spatial resolution may not be realistic. Given that it is not feasible to fit large housings with a full grid of antennas of the current size, an alternative could be to fit specific areas in the housing with antennas—for example, the feeders. This would not provide the detail in the activity level that could be recorded in the present study, but does allow one to keep track of feeder visits. Another option would be to use several line antennas to divide the overall area into different zones, and to study how often birds switch between zones. Future research could look into how well these crude measurements correlate with detailed data on activity levels in order to determine whether these crude measurements can serve as a proxy for activity in large scale production environments.

Future research should furthermore look into alternative methods for attaching the RFID tags to the legs. The rubber bands used in this study were effective, i.e., the tags were well secured and not often lost, and evoked little reaction from the broilers after a short habituation period in which some pecking at the leg bands was observed, suggesting that the leg tags did not interfere with the normal behaviour of the broilers. Furthermore, no issues were observed with the rubber bands catching on objects in the environment, although it must be noted that all the objects in the pen and the pen walls had smooth surfaces. However, the leg bands had to be checked every couple of days and had to be switched to a larger size during the trial to avoid the rubber bands becoming too tight. Therefore, there would be added value to leg bands that can keep up with the growth of the broilers’ legs in order to reduce the required manual labour.

Future research should also look into the potential of using RFID recordings to determine true distances moved in more detail. The relationship between RFID and video, which seems to show a linear pattern, indicates that there is potential to use RFID recordings to estimate true distances moved. Although not studied here, the RFID system could possibly be implemented to study true distances moved of group-housed birds, which could be a valuable tool in, for example, determining threshold levels of activity for reducing leg problems.

## 5. Conclusions

It was studied here whether a passive high frequency RFID tracking system was suitable for monitoring the individual levels of activity of broilers throughout life. In 62.5% of the cases, the RFID system was in full agreement with video in terms of the location of the animals. In total, 99.2% near matches, allowing for a deviation of one antenna, were observed between RFID and video in terms of location. There were moderately strong correlations in terms of distances moved between RFID and video (r_s_ = 0.82) and between RFID and UWB (r_s_ = 0.70). This indicates that the RFID system can accurately register among-individual variation in activity. However, the absolute values of the RFID recorded distances are generally an underestimation of the true distances moved. Overall, the RFID system appears suitable for monitoring the relative activity of individual group-housed broilers and can contribute to obtaining activity measures at the individual level early, and later, in life. Main benefits of the RFID system for tracking activity, compared to, for example, UWB tracking or video observations, include its small, lightweight tags that allow tracking of broilers from hatching onwards, limited manual labour and reliable identification of individuals. Data on activity levels early in life can potentially aid in identifying and predicting health, welfare and production parameters, but more research in this area is required.

## Figures and Tables

**Table 1:** Overview of bodyweights of the subjects in this study (n = 36) at different ages. SEMs are rounded to round numbers.

Age	Weight (Mean ± SEM)
8 days ^1^	250 g ± 4 g
15 days ^1^	620 g ± 9 g
22 days ^1,2^	1160 g ± 17 g
29 days ^1,2^	1840 g ± 25 g
34 days ^3^	2414 g ± 27 g

^1^ Ten-gram precision; leg tags included. ^2^ Weighed including UWB tags; 25 g in weight subtracted for birds with UWB tags. ^3^ Different scale used with two-gram precision; leg tags and UWB tags not included.

**Table 2:** Overview of the characteristics of the videos that were selected for the location validation study. UWB = ultra-wideband.

Video	Age of the Broilers (Days)	Time of Day (Start of Video)	Duration (mm:ss)	Average Expected Activity Level	UWB Tags Present
Video 1	1	09:48:49	06:47	Low	No
Video 2	11	10:43:32	06:49	High	No
Video 3	18	11:53:42	06:55	High	Yes
Video 4	34	11:05:11	06:59	Low	Yes

**Table 3:** Overview of the percentage of exact matches and near matches between video and RFID registrations for the four videos.

Video	Bird Age (Days)	Expected Activity	UWB	Registrations Included ^1^	Exact Matches	Near Matches ^2^
Video 1	1	Low	No	1319	72.1%	100.0%
Video 2	11	High	No	1576	80.9%	99.9%
Video 3	18	High	Yes	1463	47.4%	97.0%
Video 4	34	Low	Yes	1575	50.0%	99.9%
Total				5933	62.5%	99.2%

^1^ I.e., the number of available RFID and video locations to compare in the respective video. ^2^ Allowing for a deviation of one antenna, i.e., a registration at one of the maximum eight neighbouring antennas.

## Data Availability

The data that support the findings of this study are available from Cobb Europe but restrictions apply to the availability of these data, which were used under license for the current study, and so are not publicly available. Data are however available from the authors upon reasonable request and with permission of Cobb Europe.

## Supplementary Materials

The following are available online at https://www.mdpi.com/1424-8220/20/13/3612/s1, Table S1: Intra-observer reliability scores for every observer.
