# Supplementary material for: Assessing the Activity of Individual Group-Housed Broilers Throughout Life Using a Passive Radio Frequency Identification System—A Validation Study
Source: Sensors (Basel). 2020 Jun 27;20(13):3612. doi: 10.3390/s20133612 (PMC7374484; doi:10.3390/s20133612)
Supplement: Supplementary file 1 [file sensors-20-03612-s001.pdf]

Table S1. Intra-observer reliability scores for every observer.

Observer	Subjects	Raters	Kappa	Z	p-value
1	95	2	0.988	29.3	<0.001
2	96	2	0.977	29.6	<0.001
3	95	2	0.929	27.5	<0.001
4	94	2	0.753	23.6	<0.001
5	83	2	0.879	24.9	<0.001
6	95	2	0.953	28.5	<0.001
7	95	2	0.929	27.7	<0.001
8	92	2	0.951	27.5	<0.001
9	96	2	0.907	27.4	<0.001
10	93	2	0.952	27.8	<0.001
11	95	2	0.976	29.1	<0.001
12	98	2	0.887	27.9	<0.001
